# Metal concentrations in hair of patients with various head and neck cancers as a diagnostic aid

**DOI:** 10.1007/s10534-015-9899-8

**Published:** 2015-12-11

**Authors:** Anna Wozniak, Marta Napierala, Magdalena Golasik, Małgorzata Herman, Stanisław Walas, Wojciech Piekoszewski, Witold Szyfter, Krzysztof Szyfter, Wojciech Golusinski, Danuta Baralkiewicz, Ewa Florek

**Affiliations:** Laboratory of Environmental Research, Department of Toxicology, Faculty of Pharmacy, Poznan University of Medical Sciences, 30 Dojazd Street, 60-631 Poznan, Poland; Department of Analytical Chemistry, Faculty of Chemistry, Jagiellonian University, 3 Ingardena Street, 30-060 Krakow, Poland; Laboratory of High Resolution Mass Spectrometry, Regional Laboratory of Physicochemical Analysis and Structural Research, Faculty of Chemistry, Jagiellonian University, 3 Ingardena Street, 30-060 Krakow, Poland; Department of Otolaryngology and Laryngological Oncology, Poznan University of Medical Sciences, 49 Przybyszewskiego Street, 60-355 Poznan, Poland; Institute of Human Genetics, Polish Academy of Sciences, 32 Strzeszynska Street, 60-479 Poznan, Poland; Department of Head and Neck Surgery, Greater Poland Cancer Centre, Poznan University of Medical Sciences, 15 Garbary Street, 61-866 Poznan, Poland; Department of Trace Elements Analysis by Spectroscopic Method, Faculty of Chemistry, Adam Mickiewicz University, 89b Umultowska Street, 61-614 Poznan, Poland

**Keywords:** Head and neck cancers, Stimulants, Hair, Necessary elements, Toxic metals

## Abstract

Head and neck cancers are one of the most frequent cancers worldwide. This paper attempts to evaluate disturbances of homeostasis of the necessary elements (calcium, magnesium, zinc, copper, iron, manganese) and changes in the levels of toxic metals (lead, cadmium, cobalt, chromium VI) in hair of patients with head and neck cancers, as well as people without a diagnosed neoplastic disease. In order to quantify the necessary elements and toxic metals, a method using ICP-MS and ICP-OES techniques had been developed and validated. The studies have shown that patients with head and neck cancer used to drink alcohol and smoked much more frequently than healthy individuals, both in the past and presently. Statistically significant differences in concentrations of average metal content in the group of patients with head and neck cancers compared to the control group were confirmed. Significant differences in metal content between the group of patients with head and neck cancers and healthy individuals were found which enabled distinguishing between the study groups. To this end, a more advanced statistical tool, i.e. chemometrics, was used. The conducted research analyses and the use of advanced statistical techniques confirm the benefits of using alternative material to distinguish the patients with head and neck cancers from the healthy individuals.

## Introduction

Head and neck cancers are one of the most frequent cancers worldwide. It is estimated that the number of new cases is 600,000 per year, of which 300,000 are fatal (Wyss et al. 2013). In terms of global prevalence, head and neck cancers rank sixth. Two-thirds of the cases are recorded in developing countries. The risk of getting a mouth cancer is the highest in Malaysia, South and Central Asia, West and South Europe, and South Africa. Laryngeal cancer most frequently affects population of Southern and Eastern Europe, South America, and Western Asia. Mean age of patients diagnosed with head and neck cancer is around 60, with men affected more frequently, in particular in the case of laryngeal cancer (Argiris et al. 2008).

It is estimated that by 2020, due to continuous growth and aging of the general population, prevalence may double up to over one million people, with mortality rates reaching as much as half a million a year (Marron et al. 2010).

Key risk factors for development of head and neck cancers are tobacco smoking and drinking spirits. Alcohol exacerbates cancerogenic processes associated with tobacco smoke as well as being a risk factor on its own (Vokes et al. 1993). Sapkota et al. (2007) have shown in their study that chewing tobacco products may lead to pharyngeal and oesophageal cancers and is a separate risk factor along alcohol and tobacco smoking. At least 75 % of head and neck cancers diagnosed in Europe, the United States, and other industrialized world regions are due to combined effect of smoking and alcohol drinking (Hashibe et al. 2009). Distinguishing harmful effects due to alcohol drinking and tobacco smoking is very difficult because heavy drinkers are also smokers and vice versa. Moreover, most of the available study papers contain very few descriptions of cases in which the patients used to either smoke or drink alcohol (Altieri et al. 2005).

Other risk factors responsible for head and neck cancers are viral infections, for instance with infections with Epstein–Barr virus associated with the nasopharyngeal cancer, and human papilloma virus (HPV) which may be the underlying factor in particular in oropharyngeal cancer (Mehanna et al. 2011).

Moreover, other factors of note, include low body mass index (BMI), risky sexual conduct, occupational exposure, incorrect mouth hygiene, too low intake of vegetables and fruit (wrong diet), long-term exposure to passive smoking and a history of cancer in the family (Mehanna et al. 2011).

Tobacco smoking and alcohol drinking are believed to be the main underlying causes of head and neck cancers; nevertheless, the effect of the patient’s diet is also important. There were many studies on the diet’s effect on the risk of developing head and neck cancers (Horn-Ross et al. 1997; Boeing et al. 2006; Bradshaw et al. 2012; Chuang et al. 2012).

Tobacco smoking, alcohol drinking, and wrong diet may disturb the levels of elements necessary for the body to function properly and increase concentrations of toxic, health-hazardous metals. Monitoring of body nutrition for necessary elements and toxic metals content is particularly important for human health. Most clinical methods used to diagnose deficiencies of necessary elements are based on blood, serum/plasma, and/or urine sample analyses. The choice of the appropriate type of test sample, however, depends on several factors such as toxicokinetics, convenience and invasiveness of the sampling procedure, and the possibility of sample contamination (Rodrigues et al. 2008).

Alternative materials are becoming increasingly popular in evaluation of the body’s exposure to necessary and toxic elements. Concentration of metals in hair reflects the average content in the body, taking into account the longer time of exposure to elements compared to other body fluids (Mehra and Juneja 2005).

Application of elemental analysis of the necessary elements and toxic metals in hair and other biological tissues is becoming increasingly popular in medical studies (including forensic), archaeology, and nutrition (Kumtabtim et al. 2011). Hair are primarily used in determination of elements and medicines content (organic substances, narcotic drugs, nicotine metabolites) (Chojnacka et al. 2006). Their analysis allows evaluation of health and body nutrition (Qayyum and Shah 2014). Owing to their multiple advantages, hair samples are widely used to assess human exposure to various contaminations (Rodrigues et al. 2008). They are a valuable source of information concerning exposure to toxic metals such as cadmium, lead, or arsenic. Many studies confirm statistically significant differences in elements content in hair between exposed and non-exposed groups (Chojnacka et al. 2010). Choice of alternative matrices allows non-invasive material sampling (Rodrigues et al. 2008).

Physiological metals perform important functions in the human body. Disturbed metabolism of these elements may lead to cell functioning impairment and, consequently, to a disease.

Elevated toxic metal levels in the body may be an evidence of an existing disease process. Exposure to such elements as lead, cadmium, cobalt or chromium (VI) may lead to disturbed functioning of the central and peripheral nervous systems, hepato- and nephro-toxicity, cardiovascular system impairments, and impaired functioning of the reproductive system. Toxic doses of the listed elements may lead to carcinogenesis, as confirmed by numerous studies (Patrick 2006; Gál et al. 2008; Soudani et al. 2010; Templeton and Liu 2010; Hordyjewska et al. 2014).

The aim of the study was to evaluate the concentration levels of physiological elements and toxic metals such as calcium, magnesium, zinc, copper, iron, manganese, cadmium, cobalt, and chromium (VI) in patients with parotid gland, oral and lingual, tonsil, nasopharyngeal, lower pharyngeal, and neck cancers. Hair was used as an alternative material for exposure analysis. The effect of smoking, drinking alcohol, and the type of diet on the level of these metals was taken into consideration.

## Materials and methods

The study group included patients of the Otolaryngology and Laryngological Oncology at Heliodor Święcicki Public University Hospital, Karol Marcinkowski University of Medical Sciences in Poznań; and the patients of the Head and Neck Surgery and Laryngological Oncology University Ward, Karol Marcinkowski University of Medical Sciences in Poznań. The patients were 63 men and 31 women with parotid gland, pharyngeal, oral, tonsil, cancers and neck tumors. The control group were 17 men and 46 women with non-malignant tumors such as toxic nodulargoiter, Graves’ disease, chronic paranasal sinusitis, otitis, tonsillitis, reactive inflammatory lymph nodes, diverticulum of the oesophagus, nasal septum distortion, polyps in the nose and paranasal sinuses, paralysis of vocal folds, and maxillary sinus cyst. The patient’s participation in the study was voluntary.

The test protocol was approved by the Bioethics Committee at Karol Marcinkowski University of Medical Sciences in Poznań, in Resolution no. 670/08 of 12 June 2008 and Resolution no. 129/11 of 3 March 2011. All procedures performed in studies involving human participants were in accordance with the ethical standards of the institutional and/or national research committee and with the 1964 Helsinki declaration and its later amendments or comparable ethical standards.

In the first stage, the patients answered questions in an author questionnaire. The biological material used in the study was patients’ hair. The material sampling and preparation for testing technique was presented in the papers by Cooper et al. (2012).

Reference materials, with certified values of the determined analytes, used for verification of the developed procedures for hair were GBW 07601 (GSH-1) NRCG (China).

Hair samples on every stage were mineralized using a high-pressure microwave system–Mars 6, CEM, Matthews, USA, equipped with high-pressure XP-1500 vessels. Average hair sample weight was 0.2 g. Concentrated nitric acid (V)–HNO_3_ suprapure in the amount of 5 ml was used as a mineralising solution. The mineralisation process was conducted according to the optimised mineralisation programme presented in Table 1.

**Table 1:** Optimal microwave-assisted digestion program for hair samples

Step	Stage 1		Stage 2		
	Time (min)	Variable microwave power	Time (min)	Use of 100 % microwave power	
Digestion	15	Increase of temperature to 180 °C	10	Hold of temperature (180 °C)	Microwave power: 1800 W
Cooling	30	Decrease of temperature to 40 °C	–	–	–

After the mineralisation process, the samples were cooled down to ambient temperature and then the accumulated nitrogen oxides were stripped. The digest solutions obtained were transferred quantitatively to 25 ml volumetric flasks and filled up to the line with water from the reverse osmosis process. The samples were held in a fridge (not longer than 2 weeks) at 5 °C, in 50 ml polyethylene test tubes.

Due to different concentration levels of elements in the test tube, two techniques were used to quantify the necessary and toxic elements:

- Quantification of metals using inductively coupled plasma optical emission spectrometry (ICP-OES)–this method was used to quantify calcium (Ca) and magnesium (Mg) with wavelengths of λ_Ca_, = 317.933 nm and λ_Mg_, = 285.213 nm, respectively.
- Quantification of metals using inductively coupled plasma mass spectrometry (ICP-MS)–analysis was performed for the remaining physiological elements and selected toxic elements. Measurements were performed for isotopes of the following elements: manganese (^55^Mn), iron (^57^Fe), copper (^65^Cu), zinc (^66^Zn), chromium (^52^Cr), cobalt (^59^Co), cadmium (^111^Cd), and lead (^208^Pb).

## Results

### Survey questionnaire analysis

Our studies have shown that in the control group most of the patients did not smoke, either in the past (31 subjects–53 %) or presently (52 persons–84 %). Neither did most respondents drink alcohol in the past (25 subjects–53 %), and 15 subjects admitted they were drinking one alcoholic beverage a week (31 %). Presently, a vast majority of patients—43 subjects (77 %) does not drink alcohol, while 13 (23 %) admitted they were drinking alcohol presently. It can be assumed that the non-consumption of stimulants by the control group subjects entailed an increased protection of the body against development of a neoplastic process.

A significant number of patients with head and neck cancers gave an affirmative answer to the questions on the use of stimulants in the survey questionnaire.

62 % of patients with parotid gland cancers smoked in the past. Statistics concerning pharyngeal cancer are radical–all patients in this group were smokers. Of the patients with mouth cancer 74 % smoked in the past, of respondents with neck cancer 70 % answered “yes”, while in the case of tonsil cancers as many as 90 % were smokers.

Our own studies on past alcohol consumption confirmed that most of the patients with head and neck cancer had used this stimulant. The poorest statistics were those for respondents with tonsil cancers as in this group all the patients had used to drink alcohol in the past, and 60 % of them had been drinking more than five alcoholic beverages a week. Of the patients in control group, most had not used alcohol in the past but the differences compared to non-drinking respondents were small. At the time of filling in the survey questionnaire, 77 % of patients in the control group responded that they did not drink alcohol, while 13 subjects (23 %) admitted they were drinking alcohol presently. Patients with neck tumor failed to answer this question, therefore we cannot tell if they are currently drinking alcohol. Although most of the patients do not drink alcohol, persons who still use this stimulant in their daily life can be found in every group.

The survey questionnaire included also questions on the type of diet. Most of the patients, both in the control group and cancer-affected group, liked to eat poultry, ham, eggs, and white cheeses the most. The least frequently eaten products were sprouts, seafood, and spinach.

### Elemental quantitative analysis

Elemental quantitative analysis was performed for hair samples from 87 patients with a diagnosed cancer: 26 with parotid gland cancer, 13 with pharyngeal cancer, 26 with oral cancer, 12 with neck tumor, and 10 with tonsil cancer.

Sixty-four hair samples from healthy individuals from among which a control group, of the same number of subjects, was randomly chosen for each group of patients.

Based on the obtained analysis results, key analytical parameters were determined for each study group separately–average concentrations of specific metals in the hair, their extent, and standard deviation. The results were collected in Table 2.

**Table 2:** Average values of analyte concentration and their statistical evaluation in the hair between groups (µg·g^−1^)

	Ca^a^	Mg^a^	Cu	Fe^a^	Zn	Mn	Co	Cr	Cd	Pb
Salivary gland cancer (n = 26)	3.38 ± 2.09^b^	0.36 ± 0.21^b^	29.34 ± 18.11	0.29 ± 0.19	129.35 ± 69.74	2.83 ± 2.11^b^	0.58 ± 1.81	1.61 ± 1.30	1.67 ± 3.12	39.38 ± 36.64^b^
	0.30–7.53	0.11–0.94	9.90–74.21	0.04–0.73	22.11–333.68	0.52–8.77	0.03–8.06	0.03–4.21	0.05–14.00	2.45–140.16
Laryngeal cancer (n = 13)	4.51 ± 5.33	0.26 ± 0.25^b^	66.74 ± 57.80	0.27 ± 0.28	264.29 ± 183.13	2.63 ± 2.44	0.20 ± 0.28	8.83 ± 27.80	0.28 ± 0.29	3.23 ± 1.74^b^
	0.71–19.39	0.04–0.90	13.30–210.11	0.01–0.89	60.52–631.92	0.31–8.30	LOD –0.87	0.08–101.00	0.02–1.05	0.87–6.82
Oral cancer (n = 26)	1.49 ± 1.11^b^	0.12 ± 0.08^b^	30.73 ± 20.61	0.14 ± 0.11^b^	177.35 ± 80.17	1.95 ± 1.79^b^	0.11 ± 0.15^b^	0.82 ± 0.68	0.57 ± 0.52	8.52 ± 7.30^b^
	0.29–3.84	0.02–0.27	10.71–77.61	0.02–0.50	92.24–368.75	0.26–7.55	LOD –0.51	0.12–2.39	LOD –1.76	0.89–25.22
Neck cancer (n = 12)	3.06 ± 2.02^b^	0.60 ± 0.36	13.23 ± 5.56^b^	4.46 ± 3.54^b^	140.89 ± 107.22	1.84 ± 2.22^b^	8.54 ± 29.12	0.74 ± 0.69	0.38 ± 0.33	4.61 ± 3.98^b^
	0.47–8.38	0.10–1.26	5.53–25.67	1.24–14.96	11.12–412.44	0.40–8.58	0.01–101.00	0.02–2.82	0.05–1.26	1.37–15.86
Tonsil cancer (n = 10)	4.18 ± 2.68^b^	0.35 ± 0.25^b^	28.31 ± 23.02	0.06 ± 0.04^b^	184.37 ± 124.00	2.88 ± 2.83	0.40 ± 0.77	0.27 ± 0.29^b^	0.48 ± 0.31	6.34 ± 4.13^b^
	0.66–8.06	0.07–0.78	12.15–84.18	LOD –0.12	0.59–369.24	0.18–8.07	LOD –2.44	0.04–0.91	0.08–0.93	0.68–11.70
Controls (n = 64)	8.05 ± 7.32	1.41 ± 1.44	52.58 ± 43.19	1.13 ± 2.85	214.31 ± 163.35	10.72 ± 11.54	0.33 ± 0.47	5.79 ± 19.58	0.77 ± 0.88	2.52 ± 24.52
	0.75–0.28	0.21–7.73	7.89–189.71	0.07–0.05	14.69–852.60	0.25–4.13	LOD –2.71	0.09–101.00	LOD –3.46	2.47–96.21

^a^Concentration (mg·g^−1^)

^b^Statistically significant difference in the concentrations with regard to the control group (*p* ≤ 0.05 test, Mann–Whitney U)

*LOD* limit of detection

### Statistical evaluation

For statistical evaluation of differences between mean values of hair concentrations of elements in specific patient groups and in the control group, Shapiro–Wilk test and Kolmogorov–Smirnov test with Lilliefors correction were conducted first to test variable normal distribution. The normal distribution assumption was not met for all elements in study groups, so the non-parametric Mann–Whitney U test was used to compare mean concentrations between the two groups.

In all analyses, *p* ≤ 0.05 was the threshold level of significance. Below this value, the result was deemed statistically significant.

Results of the conducted Mann–Whitney U test indicate statistically significant differences in mean contents of the following metals in patients with cancers:

- parotid gland: calcium (Ca), magnesium (Mg), manganese (Mn), lead (Pb);
- pharyngeal: magnesium (Mg), lead (Pb);
- oral: calcium (Ca), magnesium (Mg), iron (Fe), manganese (Mn), cobalt (Co), lead (Pb);
- neck: calcium (Ca), copper (Cu), iron (Fe), manganese (Mn), lead (Pb);
- tonsil: calcium (Ca), magnesium (Mg), iron (Fe), chromium (Cr), lead (Pb).

For statistical evaluation of differences between mean values of hair concentrations of elements in specific patient groups with various types of cancers, a non-parametric analysis of variances by ranks–Kruskal–Wallis ANOVA test–was performed. Results of this analysis are presented in Table 3 (presenting only the groups for which concentrations of a given element showed a statistically significant difference, with *p* < 0.05).

**Table 3:** A post hoc analysis of differences between groups–results of Kruskal-Wallis test

Element	Compared group	*p* value^a^
Mg	SGC versus OC	0.006
	OC versus NC	0.0004
Cu	SGC versus NC	0.04
	OC versus C	0.0004
	OC versus NC	0.03
Fe	SGC versus NC	0.0000
	TC versus NC	0.0000
	LC versus NC	0.0000
	OC versus NC	0.0000
Cr	SGC versus TC	0.01
Pb	SGC versus TC	0.006
	SGC versus C	0.0000
	SGC versus OC	0.002
	SGC versus NC	0.000

Concentrations of iron (Fe) and lead (Pb) in hair allow differentiation of four pairs of patient groups, copper (Cu)–three pairs, magnesium (Mg)–two, while chromium (Cr)–only one pair of groups. Patients with a parotid gland cancer are the most distinguished of all groups.

Cluster analysis was performed to verify whether the data obtained as a result of analysis of hair sampled from patients with various head and neck cancers and healthy individuals allow a clear differentiation between these two groups. Data were subject to autoscaling prior to analysis. The data were grouped using the Ward’s method. Squared Euclidean distance was adopted as the measure of distance between the clusters. Analysis of individual dendrograms, divided into groups of patients with a specific cancer and into control group, performed on hair, distinguished two or three clusters of items, depending on the disease.

### Discriminant analysis

The next step was discriminant analysis conducted to develop a model to differentiate between patients with five cancer types and healthy individuals.

Discriminant analysis was performed using concentrations of ten elements in hair for every group. In general classification, the largest concentrations were those of iron (Fe), then lead (Pb), and magnesium (Mg), and the variables with the smallest but highly important share were copper (Cu) and zinc (Zn).

**Fig. 1:**
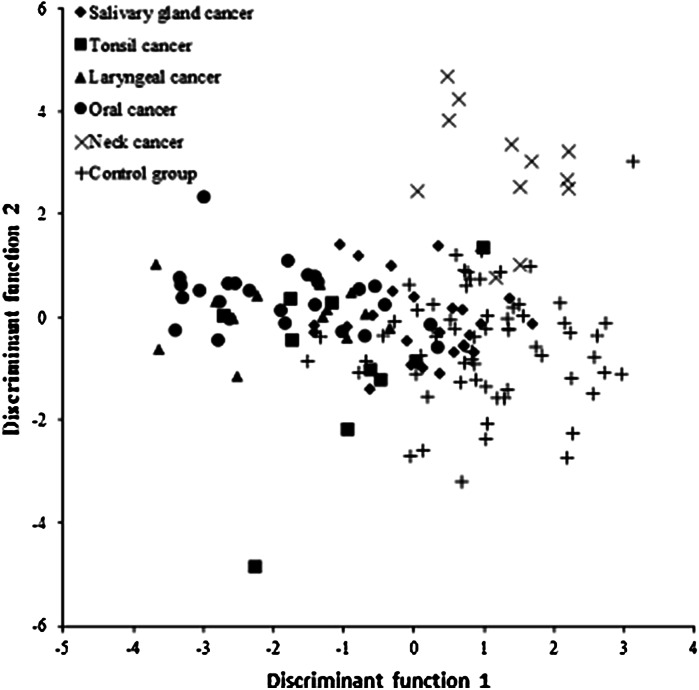
Scatterplot of the two canonical discriminant functions for metal concentrations in hair of cancer patients and control group

Five discriminant functions were developed for discrimination of six groups. Their interpretation and relevance assessment were based on canonical analysis. Results of discriminant analysis are also presented in graphic form on Fig. 1.

A discriminant function does not distinguish clearly all the groups but allows distinguishing mainly the group of patients with mouth cancer (left side of the chart) and the control group (right side of the chart).

In the next step, classification of objects was based on the same data as during the procedure of discrimination of all groups. The designated ranking functions were calculated for every group and were used for direct classification of cases. Based on the ranking functions derived, their accuracy and relevance were tested by qualifying patients into the discussed groups. *Post hoc* classification results have shown that nearly 73 % of subjects were correctly qualified into their groups. The most accurate classification—83.3 % was obtained for the group with neck tumor, and second most accurate for healthy individuals’ group (81.3 %). The least accurate classification was obtained for the tonsil cancer group with 40 % of accurate classifications.

We decided to develop a model to differentiate between patients with parotid gland cancer and neck tumor based on analysis of results of the conducted discriminant analysis. Only three of the five elements included in the model had a relevant contribution in group discrimination. Iron (Fe) had the greatest contribution to the general classification.

**Fig. 2:**
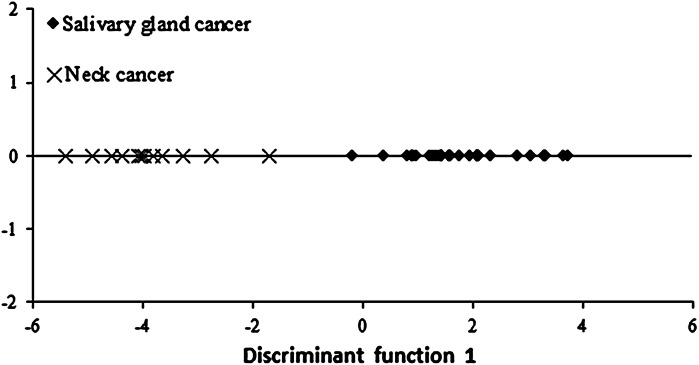
Scatterplot of the first discriminant function for metal concentrations in hair of patients with salivary gland cancer and neck cancer

In order to discriminate between two groups, one discriminant function was developed. Its interpretation and relevance assessment were based on canonical analysis. The result was standardized values of discriminant function coefficients amounting to −0.862 for iron (Fe), 0.645 for lead (Pb) and −0.570 for magnesium (Mg). Significance of the discriminant function was tested by performing a Chi square test for individual elements. The test has shown the function to be statistically significant. Results of discriminant analysis are also presented in graphic form on Fig. 2.

The discriminant function makes a clear distinction between the two groups; on the canonical function dispersion chart, the neck tumor patients are on the chart’s left side and those with parotid gland cancer–on the chart’s right side. *Post hoc* classification results have shown that it is 100 % accurate for both groups.

Another model was developed to differentiate between patients with neck tumor and pharyngeal cancer. Only two out of ten elements had a relevant contribution to group discrimination: iron and copper, while as many as eight elements fell outside the model. In this model, iron had the greatest contribution to the general classification.

**Fig. 3:**
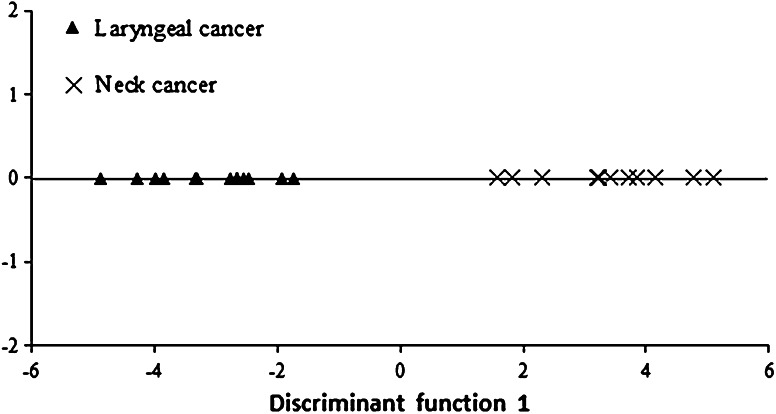
Scatterplot of the first discriminant function for metal concentrations in hair of patients with laryngeal and neck cancer

In order to discriminate between two groups, one discriminant function was developed. Its interpretation and relevance assessment were based on canonical analysis. The result was standardised values of discriminant function coefficients amounting to 1.366 for iron and 1.248 for copper. Significance of the discriminant function was tested by performing a Chi square test for individual elements. The test has shown the function to be statistically significant. Results of discriminant analysis are also presented in graphic form on Fig. 3.

The discriminant function makes a clear distinction between the two groups; on the canonical function dispersion chart, the laryngeal patients are on the chart’s left side and those with neck tumor–on the chart’s right side. *Post hoc* classification results have shown it to be 100 % accurate for both groups; it should be noted, however, that the number of cases in both groups is small.

## Discussion

Smoking and drinking spirits are the main risk factors in development of head and neck cancers. This fact has been confirmed in many research studies (Castellsagué et al. 2004; Döbrőssy 2005; Hashibe et al. 2009).

Results of the conducted studies confirmed that most of the patients with head and neck cancer had been or still is smoking. The analysis performed corroborates with the finding of Marur and Forastiere (2006) who claimed that smoking addicts are at 5–25 times higher risk of developing head and neck cancers than non-smokers. Control group patients were predominantly non-smokers.

In mid-1950s, a cause and effect relationship between increased alcohol consumption and development of a squamous cell mouth, laryngeal, pharyngeal, and oesophageal cancers has been noticed (Altieri et al. 2005). Our own studies, conducted with a survey questionnaire, have shown that majority of patients with head and neck cancer used to drink alcohol in the past.

Epidemiological studies on head and neck cancers have shown that long-term alcohol consumption shows a linear correlation with development of neoplastic lesions (Boffetta and Hashibe 2006). That is why it is so important to stop using this stimulant; according to Altieri et al. (2005), health benefits after giving up alcohol drinking have been observed already after 5 years of withdrawal, even though effects of giving up alcohol addiction are noticeable only in the long term.

The patients, both in the control group and cancer-affected group, liked to eat poultry, ham, eggs, and white cheeses the most. The least frequently eaten products were sprouts, seafood, and spinach. All these products are a rich source of vitamins and microelements. Spinach is known for its anti-cancer properties–it is abundant in potassium, iron, magnesium, phosphorus, and contains vitamin C which is a popular anti-oxidant. Seafood, in turn, contains iron, magnesium, calcium, zinc, and selenium famous for its anti-cancer properties. Sprouts, likewise, protect against cancer, with their large content of potassium, phosphorus, iron, zinc, iodine, selenium, and vitamins A, C, and B.

Many studies have shown that a greater consumption of fruit and vegetables reduces the risk of cancers developing in this body region (Li et al. 2012). Lucenteforte et al. (2009) have shown in a meta-analysis that fruit and vegetables are the most important and the most desirable diet ingredient in patients with mouth and laryngeal cancers. Of studies in Italy, 20–25 % of cancers in this body region resulted from low intake of fruit and vegetables, and this rate increased to 85–95 % when such diet was accompanied by smoking and drinking alcohol. Knowledge on the effect of milk and dairy products, as well as of coffee and tea on development of mouth and laryngeal cancers is incomplete.

There are few studies providing information on nutrition of patients with cancers. It is known that persons affected with this disease are frequently malnourished and weakened, and these conditions deteriorate during the disease. Anorexia and cachexia syndromes, common in cancer patients, entails a pessimistic diagnosis and poor quality-of-life of the patients (Hutton et al. 2006). Understanding eating preferences and habits of this patient group is necessary to develop effective recommendations to improve their health and quality of life.

Analysis of hair as alternative material has shown higher concentrations of necessary and toxic elements than reported in the references, even though these values vary among research papers (Chojnacka et al. 2006; Guo et al. 2007; Pasha et al. 2007, 2010; Afridi et al. 2011; Blaurock-Busch et al. 2014).

Analysis of calcium concentration in the control group has shown a much higher content of these elements than in the groups of patients with cancers. Statistically significant values compared to the control group without cancers (*p* ≤ 0.05, Mann–Whitney U test) were recorded for parotid gland cancer, mouth cancer, neck tumor, and tonsil cancer. The highest calcium values among the cancer groups were noted in patients with laryngeal and tonsil cancers, and the lowest in patients with mouth cancer. For magnesium, hair concentrations in control group patients were higher than in patients with head and neck cancers. Statistically significant differences were noted for laryngeal and tonsil cancers.

Unkiewicz-Winiarczyk et al. (2009) in their paper recorded lower values of calcium and magnesium in smokers’ hair. In our own studies, most cancer patients had been once addicted to nicotine. Low levels of these elements might have resulted from lower intake of these elements in the diet. This is because smokers are frequently affected with distorted sense of taste (dysgeusia) (Unkiewicz-Winiarczyk et al. 2009). Patients with head and neck cancers also have an impaired ability to consume foods due to lack of hunger, distorted taste, loss of appetite, dry mouth, pain in the oral cavity, infections, and difficulties in swallowing and chewing food. Guo et al. (2007) also noticed higher values of calcium and magnesium in hair of control group patients compared to patients with prostate cancer. Pasha et al. (2007), in turn, noticed that cancer patients had higher values of these two necessary elements in hair than healthy individuals.

For copper, statistically significant differences compared to the control group were found in hair of patients with neck tumors, while for iron these differences were observed in patients with mouth or tonsil cancer, and with neck tumor. In the control group, iron levels were higher than in all of the cancer groups while hair copper concentration was highest in patients with laryngeal cancer. Patients with mouth, parotid gland, tonsil cancers and with neck tumor had lower copper concentrations than patients in the control group.

Concentration levels varied between studies by other authors conducting measurements of element contents in hair. Joo et al. (2008) have shown that patients with mammary gland cancers had lower iron and copper levels than control group subjects. Except for copper levels in patients with laryngeal cancers, our studies have also confirmed these correlations. A study by Al-Farsi et al. (2013) has also shown higher copper levels in the control group, while higher iron values had been recorded for autistic children. Patients with gastrointestinal cancers (Pasha et al. 2010) and lung cancers had higher iron and copper values than the control group subjects. Same correlations have been found in the study by Pasha et al. (2007).

In our study, the highest hair zinc concentration values have been recorded for laryngeal cancers. In patients with mouth, parotid gland, tonsil cancers and neck tumors, this metal’s concentration values were lower than in the control group subjects, although no statistically significant differences have been recorded for any of the cancer study groups compared to the control group.

Unkiewicz-Winiarczyk et al. (2009) have also shown reduced zinc concentrations in smokers’ hair. Assuming that hair zinc level reflects its content in the body, one can conclude that smoking cigarettes reduced this level. Insufficient zinc supply and improper absorption of this element may be the cause of its insufficient body content. It has also been shown that cadmium contained in tobacco smoke has an antagonistic effect on zinc which may lead to zinc level reduction (Unkiewicz-Winiarczyk et al. 2009). In patients with prostate and lung cancers, Karimi et al. (2012) as well as Qayyum and Shah (2014) have also shown higher zinc levels in the control group than in the cancer-affected group. Pasha et al. (2010), in turn, have shown higher zinc values in patients with gastrointestinal cancers.

The last of the tested necessary element was manganese. Statistically significant differences have been shown in patients with parotid gland and mouth cancers and neck tumors compared to the control group. The highest manganese values have been recorded in control group subjects. Leung and Huang (1997) reached similar conclusions after measuring the content of 11 elements in the hair of nasolaryngeal cancer patients. The patients were divided into four groups–control group, newly diagnosed group, patients treated for 3 months, and for 6 months. The study found lower manganese values in newly diagnosed patients compared to the control group. The authors noticed, however, that elements’ contents begin to decrease and gradually reach the same levels as metals in control group subjects (Leung and Huang 1997).

In our study, we have also tested toxic elements concentrations in hair of patients with head and neck cancers and of control group subjects. Elevated levels of toxic elements may be due to a large number of physiological disorders (Pasha et al. 2007).

Cobalt is generally known to be a component of vitamin B12. In higher concentrations, however, it may be cancerogenic (Qayyum and Shah 2014). Inhaling cobalt in large amounts may cause damage, mainly to the lungs, leading to asthma, pneumonia, or lung cancer. Cobalt’s cancerogenic properties are associated with its ability to inhibit repair mechanisms and to cause DNA damage (Qayyum and Shah 2014). The highest concentration of this metal has been noted in patients with neck tumor, the lowers in patients with mouth cancer–the difference was statistically significant compared to the control group. Pasha et al. (2007) in their study have shown higher cobalt values in cancer patients compared to the control group. Leung and Huang (1997), in turn, have shown lower concentrations of this metal in patients with nasolaryngeal cancers than in the control group.

Inhaling large amounts of chromium (VI) may lead to nasal ulcers, rhinitis, and breathing problems such as asthma, cough, or dyspnoea. Long-term exposure to this element may cause liver and kidney damage, blood circulation disorders, and nervous system disorders (Abdulrahman et al. 2012). Chromium is another element with cancerogenic properties. Experimental studies have confirmed that chromium stimulates production of oxygen radicals which damage the DNA (Pasha et al. 2010). In our studies, the highest chromium (VI) concentration was obtained for laryngeal cancer patients, and the lowest level of this elements, showing a statistically significant difference compared to the control group, was found in tonsil cancer patients. Study by Qayyum and Shah (2014) has shown higher chromium concentrations in control group subjects than in lung cancer patients. Pasha et al. (2007), in turn, have found a reverse correlation–cancer patients had higher levels of this metal than healthy individuals in the control group.

Results obtained by Åkesson et al. (2008) confirmed the hypothesis that cadmium may affect estrogen levels and thus increase the risk of hormone-dependent cancers. Smokers and people living in polluted areas have higher blood and urine cadmium levels, with smoking making the level of this element many times higher than in non-addicted individuals (Afridi et al. 2011). Studies have confirmed that drinking alcohol in combination with smoking leads to increased absorption and accumulation of cadmium in all body tissues (Afridi et al. 2011).

In parotid gland cancer patients, the most elevated cadmium concentration has been found, while the lowest has been noted in the laryngeal cancer group. No statistically significant differences have been recorded for this metal compared to the control group. Blaurock-Busch et al. (2014) in a study conducted on patients with various cancers have shown similar cadmium values in patients and in the control group; nevertheless, the highest concentrations have been noted in mammary gland cancer patients. Qayyum and Shah (2014), on the other hand, have shown higher cadmium levels in lung cancer patients than in the control group.

Lead is a very toxic metal and may enter the body in many ways. It is capable of accumulation, leading to damages of many organs and systems (Abdulrahman et al. 2012). It is considered a mutagenic element, among other reasons due to its lipid peroxidation enhancing effect (Blaurock-Busch et al. 2014). Lead may also be found in high concentrations in blood and urine of smokers (Afridi et al. 2011). Epidemiological studies have also shown a potential relationship between exposure to lead compounds at work and incidence of lung cancers (Qayyum and Shah 2014).

A large discrepancy between lead concentrations in various groups of patients and the control group has been found. The most elevated level has been noted in patients with parotid gland cancer, while in laryngeal cancer patients this level was much lower. Interestingly, statistically significant differences compared to the control group without cancers have been recorded in all groups of patients with head and neck cancers (*p* ≤ 0.05, Mann–Whitney U test). In studies by other authors, lead concentration values likewise varied. Qayyum and Shah (2014) have shown higher lead levels in lung cancer patients than in the control group. Concentration levels in the study of the above authors were similar to results of our studies. Pasha et al. have determined similar values for lead to those presented in the dissertation–they were higher in cancer patients. Blauroch-Busch et al. (2014), on the other hand, compared the values of lead and other elements in various types of cancer. The highest values have been shown in patients with mammary gland cancers and they were significantly higher than in patients with uterine cancer. Comparing all cancer cases in patients, the authors have shown the patients to have higher lead concentration levels than healthy individuals being the control group.

Statistical analysis applied, extended by an additional research tool–chemometrics, is one of techniques allowing better differentiation between the study groups; other measurement methods need to be used, however, in order to validate the measurements performed. Statistical analyses should be combined with clinical trials as such approach only can yield more reliable results. Routine application of this model would require harmonization of research techniques and using more numerous study groups. This could facilitate and accelerate the process of diagnosing the patients and help reduce the morbidity.

## Conclusion

Quantification of the necessary elements (calcium, magnesium, zinc, copper, iron, manganese) and toxic metals (lead, cadmium, cobalt, and chromium (VI) in hair helped us develop and streamline an analytical procedure which may be effectively used in clinical and toxicological studies to quantify these elements in very low concentration ranges.

The conducted research analyses and the use of advanced statistical techniques confirm the benefits of using alternative material to distinguish the patients with head and neck cancers from the healthy individuals.

## Abbreviations

ICP-OES: Inductively coupled plasma optical emission spectrometry
ICP-MS: Inductively coupled plasma mass spectrometry
